# From black particles to molecular proof: PD-associated peritonitis by *Daldinia eschscholtzii*

**DOI:** 10.1016/j.mmcr.2026.100773

**Published:** 2026-02-13

**Authors:** Patcharee Tedchai, Oranan Thamvichitkul, Charuwan Tiboonbun, Wissanu Srinu, Talerngsak Kanjanabuch

**Affiliations:** a Dialysis Unit of Sisaket Hospital, Sisaket, Thailand; b Dialysis Unit of Kantharalak Hospital, Sisaket, Thailand; c Medicine department of Sunpasitiprasong Hospital, Ubonratchathani, Thailand; d Center of Excellence in Kidney Metabolic Disorders, Faculty of Medicine, Chulalongkorn University, Bangkok, Thailand; e Division of Nephrology, Department of Medicine, Faculty of Medicine, Chulalongkorn University, Bangkok, Thailand; f Peritoneal Dialysis Excellent Center, King Chulalongkorn Memorial Hospital, Bangkok, Thailand; g Trinity Center-ISN Interventional Nephrology Training Center, Bangkok, Thailand

**Keywords:** *Dematiaceous mold*, *Daldinia eschscholtzii*, *Fungal peritonitis*, *Galactomannan biomarker*, *Molecular identification*, *Peritoneal dialysis*

## Abstract

Fungal infection in peritoneal dialysis (PD) is rare but devastating, especially with filamentous molds. We report a 60-year-old man on continuous ambulatory PD who presented with black particulate material in the PD transfer set and mild abdominal discomfort without cloudy effluent. PD effluent showed low-grade neutrophilic inflammation, and microscopy revealed dematiaceous septate hyphae. A slow-growing pigmented mold, initially misidentified as *Cladosporium*, was definitively identified as *Daldinia eschscholtzii* by multi-locus sequencing, supported by galactomannan positivity. This first report of PD-associated peritonitis due to *D. eschscholtzii* positions black intraluminal particles as an early visual warning sign of PD catheter-associated dematiaceous mold infection, underscoring the need for integrated mycological diagnostics before overt peritonitis develops.

## 1 Introduction

Peritoneal dialysis (PD)–associated peritonitis remains a leading cause of technique failure and adverse clinical outcomes. Although fungal peritonitis is relatively uncommon, it carries disproportionate morbidity, with strong associations with permanent discontinuation of PD, and excess mortality [1]. Current International Society for Peritoneal Dialysis (ISPD) guidelines recommend prompt catheter removal and prolonged antifungal therapy once fungi are identified; however, these recommendations are based largely on experience with *Candida* species and overt peritonitis, leaving gaps in the recognition and management of early or atypical fungal disease [2].

**Fig. 1:**
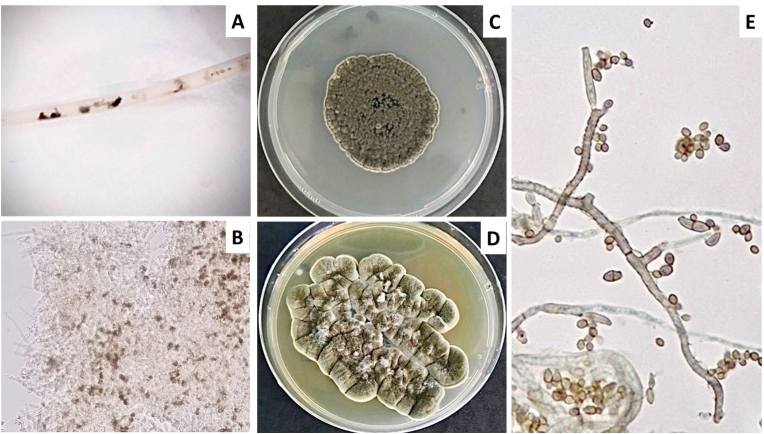
Macroscopic and microscopic features of *Daldinia eschscholtzii* isolated from the PD catheters and effluents. (A) Black particulate material adherent to the PD transfer set, representing gross intraluminal debris that prompted mycological investigation. (B) Potassium hydroxide preparation of material scraped from the transfer set demonstrating septate hyphae with focal dematiaceous pigmentation embedded within granular debris, consistent with melanized fungal elements. (C) Colony morphology on Sabouraud dextrose agar (SDA) after 9 days of incubation, showing a gray to olivaceous, compact colony with a rugose surface and well-defined margins. (D) Colony morphology on SDA after 14 days of incubation, demonstrating progressive melanization with a cerebriform, leathery appearance, characteristic of dematiaceous molds. (E) Lactophenol cotton blue preparation ( × 1000) revealing septate hyphae with poorly differentiated asexual elements and absence of organized conidiophores, a morphology consistent with *Daldinia* spp. and explaining initial misidentification as *Cladosporium* spp. in routine culture.

Accumulating evidence indicates that filamentous molds—particularly dematiaceous (melanized) fungi—represent an under-recognized subset of PD-related fungal infections, especially in tropical settings. National surveillance data demonstrate that non-hyaline molds account for approximately 15% of fungal PD infections and are associated with the poorest survival outcomes [3]. *Daldinia eschscholtzii* is a wood-inhabiting dematiaceous fungus within the family Xylariaceae, historically classified under the genus *Hypoxylon* in earlier taxonomic systems and literature [4]. Human infections caused by this species are rare, and it has not previously been characterized in association with PD-related infection. We report a unique case of early PD-associated peritonitis caused by *D. eschscholtzii*, initially signaled by black particulate material, subtle abdominal discomfort, and low-grade neutrophilic inflammation, with the diagnosis confirmed by multi-locus molecular sequencing—highlighting a diagnostically challenging yet clinically significant presentation of dematiaceous mold infection in PD.

## 2 Case presentation

A 60-year-old Thai man with kidney failure secondary to long-standing hypertension had been maintained on continuous ambulatory PD (CAPD) since June 2015 (dialysate volume 1.5 L × 4 exchanges/day). He performed exchanges independently at home and had no history of diabetes mellitus, malignancy, or immunosuppressive therapy. The patient experienced a prior episode of bacterial peritonitis on October 11, 2019 caused by *Acinetobacter baumannii*, which resolved following intraperitoneal cefazolin and ceftazidime.

On January 3, 2020 (day 0), he presented for routine follow-up after noticing black particulate material within the PD catheters (Fig. 1A). He reported mild, non-progressive abdominal discomfort without fever, gastrointestinal symptoms, or cloudy PD effluent (PDE). Physical examination revealed no abdominal tenderness, guarding, or rebound. PDE analysis demonstrated a leukocyte count of 102 cells/μL with 56% neutrophils, indicating low-grade PD-associated peritonitis. Routine bacterial cultures of the dialysate were negative. However, potassium hydroxide preparation of material from the transfer set revealed septate hyphae with focal dematiaceous pigmentation (Fig. 1B), prompting further fungal investigation.

Fungal culture from the transfer set yielded a slow-growing, darkly pigmented mold, while concurrent dialysate cultures showed no growth. On sabouraud dextrose agar (SDA, Oxoid, Hampshire, UK), colonies became gray to olivaceous with a rugose, cerebriform surface by day 9 of incubation (Fig. 1C), progressing to a more compact, leathery, and intensely pigmented morphology by day 14 (Fig. 1D). Microscopic examination demonstrated septate hyphae with poorly differentiated asexual elements and the absence of characteristic conidiation, a morphology consistent with dematiaceous molds and explaining the initial laboratory designation as *Cladosporium* spp (Fig. 1E). Given concern for early fungal involvement of the PD system, empirical amphotericin B deoxycholate (30 mg intravenously once daily for 14 days) was initiated.

Definitive identification was performed at a reference laboratory. Broad-range fungal polymerase chain reaction (PCR) assays targeting the internal transcribed spacer (ITS) and large subunit (LSU) regions were positive from the transfer set specimen. Multi-locus sequencing consistently identified *Daldinia eschscholtzii*, with high concordance across loci: LSU (100% coverage, 99.8% identity; GenBank MK981541.1), ITS (100% coverage, 100% identity; MW659100.1), beta-tubulin (β-tubulin) (100% coverage, 100% identity; PV085202.1), and RNA polymerase II second largest subunit (RPB2) (100% coverage, 99.6% identity; MK625012.1). Sequencing of the 18S ribosomal RNA (18S rRNA) gene showed lower discriminatory power, supporting genus-level assignment.

Serum and dialysate galactomannan (GM) assays were positive, with optical density indices of 0.84 and 0.77, respectively. Loop-mediated Isothermal Amplification (LAMP) [5,6] assays were positive in the transfer set and dialysate specimen. Antifungal susceptibility testing, performed according to Clinical and Laboratory Standards Institute (CLSI) M38 methodology [7] and interpreted using European Committee on Antimicrobial Susceptibility Testing (EUCAST) epidemiological cutoff values extrapolated from *Aspergillus flavus* [8], demonstrated low minimum inhibitory concentrations (MICs) for amphotericin B (1 μg/mL) and isavuconazole (2 μg/mL), with elevated MICs for multiple azoles. Despite the absence of severe clinical manifestations, the patient was transitioned from PD to hemodialysis (HD) on day 10. He remained clinically stable with no recurrence of fungal infection.

Environmental exposure history revealed ongoing agricultural work during CAPD, frequent contact with soil and decaying plant material, habitual use of firewood for warmth during dialysate exchanges, and a recent episode of dialysate bag leakage prior to symptom onset—providing a plausible source of contamination, as *Daldinia* species are saprophytic dematiaceous fungi commonly associated with decaying wood.

## 3 Discussion

This case broadens the spectrum of PD-associated fungal infection by documenting involvement caused by *Daldinia eschscholtzii*, a dematiaceous mold traditionally regarded as an environmental saprophyte and only rarely implicated in human disease. To our knowledge, *D. eschscholtzii* has not previously been reported in association with PD-related infection. Definitive species identification was established by multi-locus molecular sequencing with high query coverage and identity across ITS, LSU, β-tubulin, and RPB2 loci, thereby minimizing the risk of misclassification—an important consideration given the well-recognized morphologic overlap between dematiaceous fungi and *Cladosporium*-like species in routine laboratory practice [4,9].

Clinically, this case is distinguished by a subtle and non-classical presentation, characterized by mild abdominal discomfort, borderline neutrophilic PDE inflammation, and absence of cloudy PDE. This phenotype contrasts with the fulminant manifestations typically associated with *Candida* peritonitis and highlights a diagnostic gray zone in which early fungal PD-system involvement may precede overt peritonitis, particularly with low-virulence pathogens. Surveillance data suggest that mold-associated PD infections may initially present with intraluminal colonization or catheter dysfunction before marked leukocytosis develops [3]. Recognition of this early phase is clinically important, as delayed diagnosis and retention of colonized PD catheters are strongly associated with adverse outcomes in fungal peritonitis [[9], [10], [11], [12]].

Although fungal catheter colonization may be considered in such presentations, several findings in this case support true infection. The patient fulfilled the ISPD 2022 diagnostic criteria for peritonitis, with new abdominal discomfort and an effluent leukocyte count of 102 cells/μL with 56% polymorphonuclear leukocytes—meeting two required criteria despite negative routine culture [2]. The ISPD guidelines recognize that culture-negative episodes satisfying clinical and cellular criteria should be classified as peritonitis [2]. Importantly, multiple independent fungal-specific modalities were concordantly positive: dialysate GM index (GMI 0.77), positive LAMP, and PCR targeting the ITS directly from dialysate. Although Sanger sequencing was not performed on the primary dialysate PCR product, multi-locus sequencing of the cultured PD catheter isolate definitively identified *D. eschscholtzii*. The convergence of neutrophil-predominant pleocytosis, fungal antigen detection, nucleic acid amplification, and species-level molecular confirmation supports biologically active peritonitis rather than incidental colonization.

The macroscopic and microscopic findings further reinforce this interpretation. The black particulate material corresponded to progressively melanized colonies with rugose morphology on SDA and septate hyphae lacking diagnostic conidiation on microscopy—features characteristic of dematiaceous molds [4,13]. Melanin deposition, evident as dark intraluminal debris, is not merely a morphologic hallmark but a virulence-associated factor that enhances environmental persistence and promotes biofilm-like growth on indwelling devices [14,15], providing a plausible mechanism for early catheter-associated invasion.

From a diagnostic standpoint, this case underscores the limitations of culture-based methods alone in detecting mold-associated PD infections. Routine cultures were negative or initially misleading, whereas molecular assays and fungal biomarkers provided early and convergent evidence of mold involvement. GM positivity has been reported more frequently in mold-associated fungal peritonitis than in yeast infections and may serve as a useful adjunctive marker [3,[16], [17], [18], [19]]. The integration of microscopy, molecular sequencing, and biomarker testing illustrates a pragmatic diagnostic strategy for atypical or fastidious fungal pathogens.

Environmental exposure further strengthens biological plausibility. *Daldinia* species are associated with decaying wood and plant material, and the patient's agricultural activity, soil contact, firewood exposure, and recent dialysate bag leakage represent credible contamination pathways [4]. Such exposure patterns are consistent with the epidemiology of dematiaceous mold infections in tropical settings [15].

Therapeutically, the antifungal susceptibility profile—relative susceptibility to amphotericin B with elevated MICs to multiple azoles—differs from limited reports of *Daldinia* isolates from non-PD specimens. This finding reinforces the importance of isolate-specific susceptibility testing for rare molds and supports guideline-concordant amphotericin B–based regimens in mold-associated PD infections [2]. Although this patient did not develop fulminant fungal peritonitis, the decision to discontinue PD and transition to HD aligns with evidence demonstrating markedly increased mortality when fungal-colonized PD catheters are retained [3].

In conclusion, this case identifies *D. eschscholtzii* as an emerging dematiaceous mold capable of causing early PD-associated peritonitis with subtle inflammatory features. It underscores that fungal peritonitis may evolve along a continuum from initial catheter involvement to invasive disease, and highlights the importance of integrating clinical criteria, inflammatory indices, molecular diagnostics, and environmental exposure in recognizing atypical presentations.

## CRediT authorship contribution statement

**Patcharee Tedchai:** Writing – review & editing, Writing – original draft, Conceptualization. **Oranan Thamvichitkul:** Data curation. **Charuwan Tiboonbun:** Data curation. **Wissanu Srinu:** Writing – review & editing. **Talerngsak Kanjanabuch:** Writing – review & editing, Validation, Supervision, Conceptualization.

## Consent

Written informed consent was obtained from the patient or legal guardian(s) for publication of this case report and accompanying images. A copy of the written consent is available for review by the Editor-in-Chief of this journal on request.

## Funding source

This research was supported by Genomics Thailand, the Health Systems Research Institute (HSRI), Thailand [Grant No. 68-109], and the Thailand Science Research and Innovation Fund, Chulalongkorn University, Thailand [Grant Nos. HEA_FF_68_018_3000_002 and HEA_FF_69_123_3000_012].

## Declaration of competing interest

TK has received consultancy fees from VISTERRA and AstraZeneca as a country investigator, a research funding from the National Research Council of Thailand. He has also received speaking honoraria from AstraZeneca, Alexion Pharmaceuticals Inc., Fresenius Medical Care, and Baxter (Vantive) Healthcare. The remaining author declared no commercial or financial relationships that could be construed as potential conflicts of interest.
